# Discovery of Teneurins

**DOI:** 10.3389/fnins.2019.00230

**Published:** 2019-03-19

**Authors:** Stefan Baumgartner, Ron Wides

**Affiliations:** ^1^Department of Experimental Medical Science, Faculty of Medicine, Lund University, Lund, Sweden; ^2^The Mina and Everard Goodman Faculty of Life Sciences, Bar-Ilan University, Ramat Gan, Israel

**Keywords:** teneurin, ten-m, ten-a, TENM, ODZ, latrophilin, type II transmembrane protein, *Drosophila*

## Abstract

Teneurins were first discovered and published in 1993 and 1994, in *Drosophila melanogaster* as *Ten-a* and *Ten-m.* They were initially described as cell surface proteins, and as pair-rule genes. Later, they proved to be type II transmembrane proteins, and not to be pair-rule genes. *Ten-m* might nonetheless have had an ancestral function in clock-based segmentation as a Ten-m oscillator. The turn of the millennium saw a watershed of vertebrate Teneurin discovery, which was soon complemented by Teneurin protein annotations from whole genome sequence publications. Teneurins encode proteins with essentially invariant domain order and size. The first years of Teneurin studies in many experimental systems led to key insights, and a unified picture, of Teneurin proteins.

## Fly Teneurins Were First Described as Cell Surface Proteins, and as Pair-Rule Genes

### Discovery of the Teneurins

**FIGURE 1:**
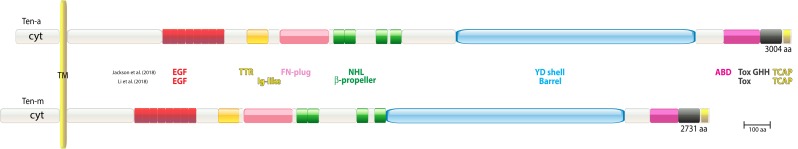
Domain structure of *Drosophila* teneurins. Only the major isoforms are shown. Domain structures are depicted according to the crystallization data of Jackson et al. (2018) and the cryo-EM data of Li et al. (2018), as they were identified and are drawn to scale. EGF, epidermal growth factor repeat; TTR, transthyretin; FN, fibronectin; NHL, NCL, HT2A Lin41; YD, YD-repeat motif; ABD, antibiotic binding domain; Tox GHH, Tox GHH fold (Zhang et al., 2012); TCAP, Teneurin C-terminal-associated Peptides; Ig, immunoglobulin.

The teneurins were discovered in the early 1990 when one of us (SB) tried to find the tenascin-C homologue in *Drosophila*. Tenascin-C is a six-armed extracellular matrix (ECM) molecule which displays many functions during development, morphogenesis and tissue homeostasis (Midwood et al., 2016). Since the *Drosophila* genome harbors a solid stock of basement membrane and other important ECM molecules (Broadie et al., 2011), it seemed conceivable to search for a *Drosophila* homologue of tenascin-C using PCR and degenerate primers. The tenascins are composed of several domains that appear in a repetitive manner such as the tenascin-type of EGF repeats or the fibronectin-type III (FN III) repeats. The carboxy terminus harbors a globular fibrinogen domain. Since all these above mentioned domains were found as parts of other *Drosophila* proteins, the question was which domain-specific primer pair would turn out to be fruitful. Of the many primers that were used in this approach, only the EGF-like domain proved successful, leading to the detection of the first *Drosophila* tenascin-type EGF-like repeats. These were then used to screen bacterial cDNA libraries that were optimized for long cDNAs (Brown and Kafatos, 1988; Brown et al., 1989) resulting in three overlapping cDNAs of 7.3 kb in length that altogether constituted a partial sequence of what had the potential to represent the *Drosophila* homologue of tenascin-C (Baumgartner and Chiquet-Ehrismann, 1993). The deduced amino acid (aa) sequence showed the presence of eight tenascin-type EGF repeats (Figure 1), as was the case in vertebrate tenascin-C (Midwood et al., 2016). At the amino terminus, a hydrophobic stretch of amino acids reminiscent of a signal or a transmembrane domain was found. C-terminally of the EGF-like repeat, an additional 100 aa were found that did not show any resemblance to FN III repeats, but soon the protein would run into a stop codon, leaving 4.3 kb of a putative 3^′^ untranslated region (UTR). Based on the deduced sequence information, the isolated composite cDNA was proposed to code for a 782 aa secreted protein and was subsequently called Ten-a (tenascin accessory) (Baumgartner and Chiquet-Ehrismann, 1993). In retrospect, the published Ten-a aa sequence from 1993 comprised only a partial sequence. This became also evident from comparing the transcript size on a Northern analysis which showed two large transcripts of 11 and 13 kb, respectively, which were developmentally regulated (Baumgartner and Chiquet-Ehrismann, 1993). The discrepancy of the length deduced from available cDNA and the actual transcript size by Northern analysis was attributed to an unusually long 5^′^ untranslated region (UTR) which later turned out not to be true. Indeed, it would take years to realize that the protein was indeed much larger (Fascetti and Baumgartner, 2002), because its coding part extended considerably in the carboxy terminal direction. This carboxy extension was also confirmed by the advent of the fully sequenced *Drosophila* genome (Adams et al., 2000).

(Baumgartner and Chiquet-Ehrismann, 1993) also showed a zoo blot equipped with DNA from *Drosophila*, leech, zebrafish, chicken, mouse, and human origin, as probed with chicken tenascin-C EGF sequences under low stringency. The blot revealed that the majority of genomes analyzed showed cross-hybridizing bands. These findings immediately opened the avenue for further quests/searches for tenascin-type EGF-like sequences not only in *Drosophila*, but later also in higher organisms (Mieda et al., 1999; Minet et al., 1999; Oohashi et al., 1999; Rubin et al., 1999; Feng et al., 2002).

The *Drosophila* lane in the zoo blot contained several cross-hybridizing bands, two of which could readily be ascribed to *Ten-a*. The *Drosophila* lane, however, revealed further unidentified bands, hence the hunt for further tenascin-EGF-like sequences was continued. To this end, one of us (S. B.) used a *Ten-a* EGF-like repeat probe and screened *Drosophila* genomic libraries under low-stringency conditions (McGinnis et al., 1984). Several cross-hybridizing phages were isolated that all mapped to a new locus (Baumgartner et al., 1994). Subsequently, overlapping cDNAs were isolated from this locus and were assembled. These cDNA clones covered two slightly smaller transcripts compared to *Ten-a*, 10.5 and 11.5 kb in size, respectively. Due to the fact that the protein encoded by the transcript of this new locus was apparently larger than that of Ten-a, this gene was termed *Ten-m* (*tenascin major*) (Baumgartner et al., 1994). At the time, it was proposed that the gene encoded a large secreted proteoglycan ECM molecule. Ten-a and Ten-m proteins’ structures and domains, as realized in 2018 terms (as described below), can be seen in Figure 1.

One of the two Teneurins was independently discovered in *Drosophila melanogaster* via an alternative approach: (*Ten-m*, as “*odd Oz*” by RW), in Levine et al. (1994). In 1990, a screen was carried out to uncover novel fly tyrosine kinase substrates of previously unknown classes. *Drosophila* proteins were highly immunopurified on an anti-phosphotyrosine antibody column, and the resulting phospho-protein collection was used to raise a bank of monoclonal antibodies. One of these specific monoclonals was directed against a greater than 300 kD protein that was later given the name Odd Oz (Odz, now Ten-m) (see Figure 1). That monoclonal was used for expression cloning of the Odz/Ten-m’s 11 kb transcript from an embryonic cDNA library (Zinn et al., 1988). Further mapping led to genomic cloning, chromosome mapping, mutant identification, and expression and phenotype characterizations (Levine et al., 1994). Two hydrophobic stretches in the predicted protein were interpreted as: (1), a signal peptide before a series of EGF-like repeats, followed by (2), a post-EGF transmembrane domain. The type-I transmembrane model was anchored by placement of the EGF-like repeats extracellularly. Yet this type-I model was also influenced by biases based on the phospho-tyrosine protein screen and consensus phosphorylation site motifs of the time. In fact, the second predicted “transmembrane” domain assignment was incorrect, and the assigned “signal peptide” sequence is the protein’s true transmembrane stretch. Odz/Ten-m is instead a type-II transmembrane protein, as all further Teneurins proved to be (see below).

### Expression of the Founding Teneurins in *Drosophila*

Both *Ten-a* and *Ten-m* genes were extensively analyzed with respect to their expression patterns during early *Drosophila* embryogenesis (Baumgartner and Chiquet-Ehrismann, 1993; Baumgartner et al., 1994; Levine et al., 1994; Fascetti and Baumgartner, 2002; Table 1). Predominant expression of both genes was in the central nervous system (CNS). In general, the *Ten-m* gene showed far more progenitor tissue labeled. Apart from its prominent CNS expression, *Ten-m* was found in the future tracheal cells, heart cells, lymph gland and hemocytes. Hence, the expression profile demanded further claims to call it “major.”

**Table 1:** Features of *Drosophila* Teneurins.

Gene name in *Drosophila*	Binding partner in *Drosophila*	mRNA expression in embryo	Localization of the protein in embryo	Localization of the protein in L3/adults	Overall phenotype in the embryo	Post-embryonic phenotype	Neural phenotype
*ten-a*	Ten-m	mat	MAS	ALG	NOP	CBD	CBD
		CNS	GC	AORN			
			AMC	AALPN			
			CNS (ant. commissure)				
*ten-m*	Ten-a	cBL: uniform	eGA: seven stripes	ED	SL	LL	Defective motor axon routing
	Cher	TR	VM	OS	pre-hatching		
	αSpectrin	CNS	MAS	WD	movement missing		
		LG	CNS	ALG			
		TR	LG	AORN			
		CB	TR	AALPN			
		HE					


*L3, third instar larvae; mat, maternal; cBL, cellular blastoderm; eGA, early gastrulation; TR tracheae, VM visceral mesoderm, MAS, muscle attachment sites; CNS, central nervous system; LG, lymph gland; HE, hemocytes; CB, cardioblast; GC gastric cecae; AMC antenno-maxillary complex; ALG, antennal lobe glomerulus, AORN, adult olfacory receptor neuron; AALPN, adult antennal lobe projection neuron; ED, eye disk; OS, optic stalk, WD, wing disk, CBD, *central body defect* (*cbd*) mutant phenotype; EL, embryonic lethality; SL semi-lethal; LL, larval lethality; V, viable; NOP, no obvious phenotype.*

Other studies expanded the breadth of Ten-m expression profiles. Striking expression was documented in non-neuronal imaginal disk tissues, such as ring gland expression (Harvie et al., 1998), in the in sensory and motor neuron precursors in pupae, and in adult neuronal tissues (Levine et al., 1997). Expression in the eye, and influences of upsteam genes such as Glass on Ten-m expression, were observed (Treisman and Rubin, 1996). Hematopoietic cells showed Ten-m expression, such as plasmatocytes (Baumgartner et al., 1994; Braun et al., 1997).

### *Drosophila Ten-m/odz* and Segmentation

Phenotypically for *odz/Ten-m*, multiple alleles from independent screens proved allelic, and displayed different severities of a pair-rule mutant phenotype (Baumgartner et al., 1994; Levine et al., 1994). This phenotype was very like that of *odd paired* (*opa*). Considerably later, the assigned pair-rule phenotype was instead attributed to mutant *opa* alleles in the genetic backgrounds of the *odz/Ten-m* strains (see below). On another note - in retrospect, previously created mutations in *Ten-a* existed that, appropriately, affect the fly brain and behavior. The gene *central body defective* (*cbd*), with several alleles known, had been isolated a decade before the gene was cloned and characterized (see Table 1; Heisenberg et al., 1985). The recognition that *cbd* mutations were *Ten-a* lesions occurred two decades later (Cheng et al., 2013).

In 2006, an indication that *odz/Ten-m* is not a pair-rule gene was published. Using new technologies that were developed, it was found that an entire 133 kb genomic clone covering *Ten-m* failed to rescue the attributed *odz/Ten-m* pair-rule phenotype (Venken et al., 2006). The concern arising from this finding led to a re-examination of all *odz/Ten-m* mutant lines displaying the pair-rule phenotype. Ultimately, the pair-rule phenotype proved to derive from *odd paired* (*opa*) mutations on the balancers in *odz/Ten-m* strains (Zheng et al., 2011).

The different *odz/Ten-m* mutations, and the balancers in their lines, came from separate mobilized-P-element screens (Cooley et al., 1988; Karpen and Spradling, 1992). The sources of the balancers for these screens were different. In addition, the many non-*odz/Ten-m* lines examined from these screens, with these balancers, displayed no pair-rule phenotype. The lines that were chosen to assess for *odz/Ten-m* lesions were based on genome position, and not pair-rule appearance, so phenotype was not a screening bias. Ten-m is deployed as seven stripes during late cellular blastoderm, but its mutants do not have pair-rule phenotypes. To this day, the reasons for the many co-incidences that led to the findings are still unclear. Unfortunately, a great deal of mis-directed work was subsequently carried out. A *Ten-a* maternal effect impact on segmentation was reported, then was later retracted (Rakovitsky et al., 2007; retracted 2012), despite the correct molecular data detailed there.

### *Ten-m* Might Nonetheless Have a Segmentation Role: A Ten-m Oscillator?

**FIGURE 2:**
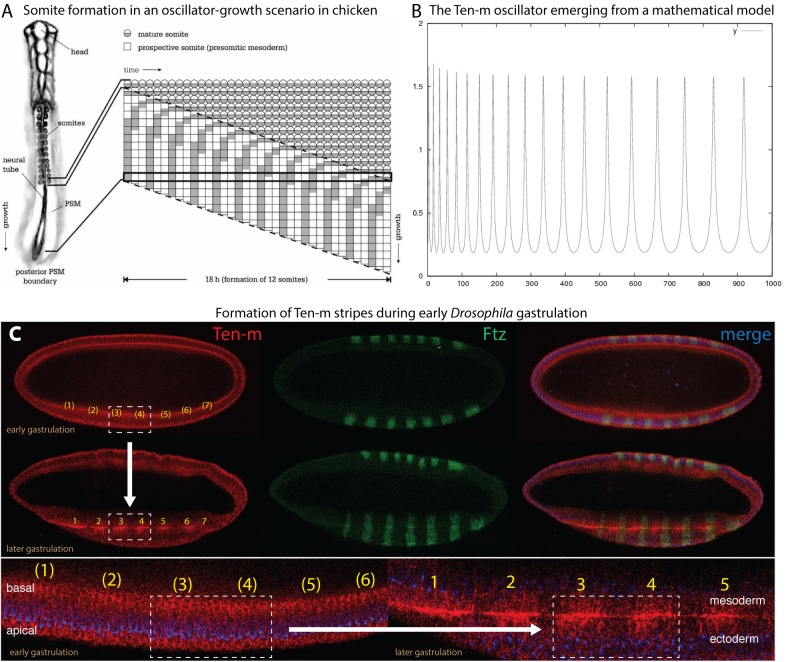
Proposed features of Ten-m as a biological oscillator. Data slightly modified from Hunding and Baumgartner (2017), see details therein. Reproduced with permission. **(A)** Somite formation in an oscillator-based system, as exemplified in chicken (Palmeirim et al., 1997). **(B)** The Ten-m oscillator as it emerges from a mathematical model. **(C)** Experimental evidence of emergence of Ten-m stripe formation during early *Drosophila* gastrulation, starting from ubiquitous Ten-m expression. Double-antibody staining reveals Ten-m in red and Fushi tarazu in green (for comparison). Top part shows the transition from ubiquitous Ten-m expression at early gastrulation to the formation of Ten-m stripes at somewhat later gastrulation (as exemplified of the boxed part comprising stripes 3 and 4 and indicated by an arrow). Bottom part shows enlargements of the formation of Ten-m stripes, again exemplified by stripe 3 and 4 formation and the boxed area. Note that Fushi tarazu (green) is already expressed in stripes from the very beginning, in contrast to Ten-m.

One aspect of *Ten-m* expression was particularly interesting because it showed its transcripts relatively uniformly expressed during cellular blastoderm, while the Ten-m protein only minutes later was detected in seven stripes (Baumgartner et al., 1994; Levine et al., 1994; Figure 2C). This observation opened the avenue for proposing a function of Ten-m as an oscillator.

In the past and first documented in the chicken *hairy* gene, it could be shown that periodically waves can arise from the posterior end of the elongating embryo. These waves move toward the anterior end where they come to a halt and add a segment during each period, as depicted in Figure 2A (Palmeirim et al., 1997). Later, a model emerged involving oscillation of the zebrafish *hairy/Enhancer of split-related* genes, *her1* and *her7* (Lewis, 2003). This model proposed that the Her protein would bind to its own promoter and inhibit its own transcription. It was then concluded that the delay would cause a biochemical oscillator because of the time difference between formation of the mRNA and the protein (Lewis, 2003). Posteriorly, cells are fed to the presomitic mesoderm (PSM) during this oscillation. When the embryo undergoes elongation, more oscillating cells are fed into the PSM that will reveal different phases. Subsequently, the cellular oscillation grows until the oscillation comes to a halt with the consequence that segmental borders will emerge. The mechanism is repeated when subsequent cells stop their oscillation. This mechanism enables that segments can be generated, starting from the anterior to the posterior.

The idea that Ten-m could be an oscillator originated from the observation that the *Ten-m* mRNA was uniformly expressed during nuclear cycle (nc) 14, but once the protein was synthesized, it started to emerge as seven stripes (Baumgartner et al., 1994; Levine et al., 1994; Hunding and Baumgartner, 2017). Since only the long nc 14 was long enough to synthesize the large primary transcript of *Ten-m* (115 kb, see Table 2) and to translate Ten-m (Prescott and Bender, 1962; Baumgartner et al., 1994), it appeared conceivable to assume that stripe formation was tightly linked to translation and to occur only during a limited time, i.e., during late nc 14. nc 14 is terminated once cellularization occurs, whereby the nuclei are wrapped by a membrane. Hence, once cellularization has taken place, signaling from the extracellular space is only possible with the help of a receptor residing at the surface of the cells.

**Table 2:** Comparison of features of *Teneurins* across phyla.

Model system	Gene name	Number of *Teneurin* genes	Nascent transcript size	Transcript size	Number of exons	Total *teneurin* gene size as% of total genome size	GenPept Accession for a representative *Teneurin* protein isoform	Protein sizes
roundworm	*ten-1*	1	26.3 kb	8.5 kb, 8.6 kb	14 (ten-1L version)	0.03	BAD91087.1, –086.1	2502 aa, 2684 aa
insect	*ten-a*	2	202 kb	11.0 kb, 13.0 kb	20	0.22	NP_001259483.1	3004 aa
	*ten-m*		115 kb	10.5 kb, 11.5 kb	9		AAF51824.2	2731 aa
ascidian	*LOC100178744*	1	48.7 kb	9.9 kb	45	0.0004	XP_018673115.1	3133 aa
chicken	*ten-1*	4	323 kb	16.9 kb	31	0.14	NP_990193.1	2705 aa
	*ten-2*		607 kb	9.5 kb	27		NP_989428.2	2802 aa
	*ten-3*		311 kb	9.6 kb	29		NP_001185466.2	2715 aa
	*ten-4*		596 kb	9.6 kb	31		NP_001341660.1	2768 aa
mouse	*ten-1*	4	901 kb	13.8 kb	32	0.13	NP_035985.2	2731 aa
	*ten-2*		1230 kb	9.7 kb	28		NP_035986.3	2764 aa
	*ten-3*		709 kb	11.0 kb	26		NP_035987.3	2715 aa
	*ten-4*		740 kb	13.5 kb	29		NP_035988.2	2796 aa
rat	*ten-1*	4	633 kb	12.4 kb	29	0.09	XP_017443608.1	2532 aa
	*ten-2*		946 kb	8.7 kb	24		NP_064473.1	2765 aa
	*ten-3*		506 kb	11.0 kb	29		NP_001162604.1	2714 aa
	*ten-4*		701 kb	8.6 kb	32		NP_001178557.1	2794 aa
human	*ten-1*	4	828 kb	12.9 kb	34	0.14	NP_001156750.1	2732 aa
	*ten-2*		1285 kb	9.6 kb	28		NP_001116151.1	2765 aa
	*ten-3*		1355 kb	10.9 kb	29		NP_001073946.1	2699 aa
	*ten-4*		788 kb	13.6 kb	31		XP_016873014.1	2794 aa


*Model systems used: roundworm, *C. elegans*; insect, *Drosophila melanogaster*; ascidian, *Ciona intestinalis*; chicken, *Gallus gallus*; mouse, *Mus musculus*; rat, *Rattus norvegicus*; human, *Homo sapiens*. aa, amino acids; kb, kilobase.*

As stated above, the *Drosophila* *Ten-m* gene encodes a large type II transmembrane protein (Figure 1) hence, it is located at the cell surface. Ten-m becomes localized to the membrane which grows from the apical side to the basal side thereby ensheathing the syncytial nuclei (Figure 2C). The large extracellular domain of Ten-m may be involved in forming homodimers, as was shown for Ten-a (Fascetti and Baumgartner, 2002) and mouse Teneurins (Feng et al., 2002; Berns et al., 2018). The dynamics of this process has properties proposed to have the potential to create a biochemical oscillator (Hunding and Baumgartner, 2017). Ten-m interaction at the membrane could lead to intracellular cleavage of Ten-m. This cytoplasmic fragment then translocates to the nucleus. As alluded to above, *Ten-m* is not transcribed in seven stripes, but rather appears fairly homogeneous along the A-P axis. The mechanism to solve this apparent discrepancy is so far not clear. However, it was proposed that the intracellular mechanism of the interplay between the protein and the membrane may lead to a spontaneous pattern-forming mechanism, as was reported from other biochemical oscillators (Hunding and Baumgartner, 2017). In fact, Ten-m fulfills most criteria of stripe formation based on a model originally described for prokaryotic cell division (Hunding and Engelhardt, 1995) and further developed by (Meinhardt and de Boer, 2001). This model has recently been recapitulated using *in vitro* data and expanded models (Loose et al., 2008).

Thus, the Ten-m oscillator is not caused by delayed translation as in the case for the zebrafish *her1/her7* genes (Lewis, 2003), but could arise from cooperative membrane binding (Hunding and Baumgartner, 2017). To enable Ten-m to function as a signaling molecule, a mechanism was proposed that would involve regulated intramembrane proteolysis (RIP) (Brown et al., 2000; McCarthy et al., 2017). Indeed, reports could show that an intracellular tail part of vertebrate Teneurin 2 protein is proteolytically cleaved off, possibly via RIP. This short peptide is then translocated to the nucleus where it represses *zic-1*, a vertebrate counterpart of *odd-*paired (*opa*), a pair-rule gene in *Drosophila* (Bagutti et al., 2003). On the other hand, ubiquitously expressed Zic-1 leads to fast degradation of the short Teneurin 2 signaling peptide. The model of Hunding and Baumgartner (2017) included thus cooperative interaction of Ten-m with the membrane, intracellular cleavage and degradation (Figure 2B).

In summary, what these data would like to suggest is that, despite the fact that *Ten-m* mutants do not show a segmental phenotype, there might be an ancestral function of *Ten-m* in clock-based segmentation. The one established by the *Notch* signaling system was probably when the insects evolved, due to the fact that Notch receptor does not show an involvement in *Drosophila* segmentation. This is where Ten-m might come in and the field is eagerly waiting for data that support this hypothesis.

## Watershed of Vertebrate Teneurin Discovery, 1998–2000, in the Pre-Vertebrate-Genome Era

**FIGURE 3:**
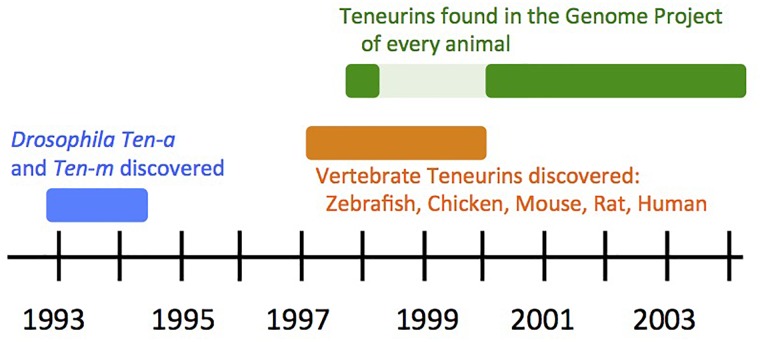
Timeline of Teneurin Discovery: the first decade.

The first publications of vertebrate Teneurin genes emerged from screens searching for other phenomena: studies of cancer rearrangements and gene changes; olfaction-related genes; and ER stress-related CHOP genes. A gene rearrangement encoding a fusion protein containing Teneurin 4 and Neuregulin 1 domains was identified in human breast tumor tissue (Schaefer et al., 1997). The resulting fusion protein contained only the pre-EGF amino-terminal portion of TENM4, but beyond ESTs, was the first harbinger of vertebrate Teneurins, as was later recognized (Wang et al., 1999). Soon thereafter, human Teneurin 1 was sequenced and named TNM (Figure 3), when it was found adjacent to the X-linked lymphoproliferative syndrome causative gene SH2D1A (Coffey et al., 1998). Teneurin 4 in mouse was uncovered in a screen for CHOP - dependent stress-induced genes, and was named DOC4 (Wang et al., 1998). Tenm4 came up twice in that screen (as DOC4 and DOC5), and was the first non-fly Teneurin to receive non-cursory treatment, with a number of pivotal observations made for the protein family as a whole. Some time later, first phenotypes for mouse Teneurin 4 were documented, when it was established that existing *l7Rn3* mice were mutants (Lossie et al., 2005). In a search based on homology to E2 cysteine rich loops of odorant receptors, rat Neurestin (Teneurin 2) was found as a novel, non-odorant receptor, protein (Otaki and Firestein, 1999a). The characterization of Teneurin 2 in Neurestin papers also contributed key observations made for the protein family as a whole (Otaki and Firestein, 1999a,b).

Meanwhile, efforts directed specifically at identifying and cloning vertebrate Teneurin by homology to the fly genes were underway in three species, and were reported in 1999 and 2000. The four paralog types in chicken were identified, Tenm1–Tenm4 (Minet et al., 1999; Rubin et al., 1999), and this work continued with many wide-ranging discoveries and publications. Four corresponding mouse paralogs were found and well characterized (Oohashi et al., 1999), and were also independently sequenced and mapped (Ben-Zur and Wides, 1999; Ben-Zur et al., 2000). Two of the four of these paralog types were also uncovered in zebrafish (Mieda et al., 1999). The rat and human Teneurin genes mentioned above were retrospectively assigned to their paralog-type number. Thus, at the end of the “pre-vertebrate genome sequence” era, five vertebrate species had been proven to bear Teneurins, with a four-copy content apparent as the common, and likely conserved, paralog complement (Figure 3).

## Analysis From the First Complete Genomes: Teneurins Form a Distinct, Animal, Family

With the completion of the *Caenorhabditis elegans* genome, a single full length Teneurin, Ten-1, was evident (Figure 3; C. elegans Sequencing Consortium, 1998). Its protein and function were characterized and described (Drabikowski et al., 2005). The *Drosophila melanogaster* genome encoded a Ten-a the length of the original Ten-m (Adams et al., 2000), as was later described (Fascetti and Baumgartner, 2002). A nematode singleton Teneurin, in contrast to a pair of paralogs, Ten-a and Ten-m, in insects, held true in the nematode *C. briggsae* (Stein et al., 2003), the mosquito *Anopheles gambiae* (Holt et al., 2002), and the silkworm *Bombyx mori* (Mita et al., 2004), genomes. Vertebrate genomes, including human (Lander et al., 2001; Venter et al., 2001), mouse (Mouse Genome Sequencing et al., 2002; Mural et al., 2002), rat (Gibbs et al., 2004), and chicken (International Chicken Genome Sequencing Consortium, 2004) validated that the four paralogs Tenm1, 2, 3 and 4 were a fixed vertebrate feature. Contemporaneously, non-vertebrate chordates, such as the ascidian *Ciona intestinalis*, proved to have a single Teneurin gene (Dehal et al., 2002). This indicated that Teneurins were quadruplicated sometime during chordate or early vertebrate evolution. A representative list of these Teneurin genes appears as Table 2. The proteins, which all maintain the same domain order, are of roughly the same size. Their protein lengths are reflected in their mature transcript sizes. Their nascent transcripts, however, are consistently of unusually large size, as is often seen for highly developmentally regulated genes. As a consequence, the Teneurin genes’ lengths occupy an “over-sized” fraction of total genome sizes (Table 2).

In contrast, Teneurins were not found in the kingdoms of plants or fungi. The earliest sequenced genomes of: the yeasts *Saccharomyces cerevisiae* (Goffeau et al., 1996) and *Saccharomyces pombe* (Wood et al., 2002); plants *Arabidopsis* (The Arabidopsis Genome Initiative, 2000) and rice (Goff et al., 2002; Yu et al., 2002), and the first other protists and fungi revealed no Teneurins. No eukaryotic homologous sequences could be found at all, outside of those to the Teneurin’s EGF-like domains. The only other Teneurin domains with homology to any proteins were *rhs* (recombination hot spot)-like elements otherwise found only in a small number of bacteria (Minet et al., 1999; Minet and Chiquet-Ehrismann, 2000).

Overall, in the first 10 years that Teneurins were studied, they were recognized as animal specific genes (Figure 3), with two paralogs in insects, and four paralogs in vertebrates. These were reviewed with an eye toward an evident ancient duplication, and an evident ancient quadruplication, in insects and vertebrates, respectively, (Tucker and Chiquet-Ehrismann, 2006; Tucker et al., 2007). These reviews also recognized that Teneurin proteins are largely invariant throughout evolution, with no domain content or order variation. For a more recent evolutionary history of the family, see the Wides article in this volume. For more recent views of Teneurins and their structure, see the Tucker (2018), and DePew et al. (2019) article, in this volume.

## From Teneurin’s First Decade: Key Insights into its Protein

The *Drosophila* Teneurin homologs were initially described as ECM molecules (Baumgartner et al., 1994), or as type I transmembrane proteins (Levine et al., 1994). The first recognition that Teneurins were in fact type II single pass transmembrane proteins came for mouse Teneurin4, when it was discovered as DOC4 (Wang et al., 1998). This was validated rigorously when all four mouse genes were sequenced, and when their extracellular portions were imaged by electron microscopy (Oohashi et al., 1999). Several studies established that Teneurins are deployed to the cell membrane as protein dimers, but their full homo- and hetero-dimerization combinatorial repertoire was first methodically shown in mouse (Feng et al., 2002). Among protein – protein interactions proven for Teneurins, perhaps the first was the fly Ten-m RGD motif interaction with integrins (Graner et al., 1998). Broader still, and iconic for Teneurin function, was the discovery of homophilic interactions in chicken (Rubin et al., 2002). While a great deal still needs to be done to nest Teneurins within a complete pathway, their homophilic, and cross-paralog-homophilic, extracellular contacts are at the heart of their signaling role. Proteolytic cleavages at many sites by many proteases are also central to varied aspects of Teneurin protein function, and have been documented since the first works published. They are too numerous to be related here, but two perhaps suggest the most important functional implications. The cleavage and release of intracellular domains, and their freedom to then enter the nucleus to impact transcription was first described in chicken (Bagutti et al., 2003; Nunes et al., 2005). The cleavage of their extreme carboxy-terminal amino acids to yield independent, biologically active TCAPs (Teneurin C-terminal-associated Peptides) occurs in many important systems (Qian et al., 2004).

Interestingly, the studies on the Teneurin domain structure were recently complemented by two reports showing data based on crystallization and cryo-EM analyses, respectively (Jackson et al., 2018; Li et al., 2018). These in principle confirmed the sequence-based data, however, they revealed that most central domains merged into a large and centrally located 200 kD superfold. They also disclosed new findings, e.g., by highlighting the NHL domain (Figure 1) as a particularly well exposed domain where homophilic interactions between teneurins were ascribed (Berns et al., 2018). Moreover, alternative splicing within the NHL domain would allow modulation of this homophilic interaction. Based on sequence comparisons, both Ten-a and Ten-m follow the domain structure that the most-recent crystallization and cryo-EM data defined. Hence, the domain structure as drawn in Figure 1 likely holds true. Evolutionarily, the 200 kD superfold was adopted as a whole structure from bacteria. This was recognized in these two papers Jackson et al. (2018), Li et al. (2018), and in Ferralli et al. (2018). Teneurin’s Latrophilin binding, and its implications, was discovered well after the first decade, and is extensively treated in other articles in this volume.

## Author Contributions

SB and RW collaborated and contributed equally to this paper.

## Conflict of Interest Statement

The authors declare that the research was conducted in the absence of any commercial or financial relationships that could be construed as a potential conflict of interest.
